# Fundus Autofluorescence and Optical Coherence Tomography Findings in Branch Retinal Vein Occlusion

**DOI:** 10.1155/2012/638064

**Published:** 2012-11-13

**Authors:** Tetsuju Sekiryu, Tomohiro Iida, Eiichi Sakai, Ichiro Maruko, Akira Ojima, Yukinori Sugano

**Affiliations:** Department of Ophthalmology, Fukushima Medical University School of Medicine, Fukushima 960-1295, Japan

## Abstract

*Purpose*. To describe the findings of fundus autofluorescence (FAF) and optical coherence tomography (OCT) in patients with branch retinal vein occlusion (BRVO). *Methods*. In this institutional, retrospective, observational case series, FAF was evaluated in 65 eyes with BRVO in 64 consecutive patients and compared with visual acuity, OCT findings, and other clinical observations. *Results*. Five types of autofluorescence appeared during the course of BRVO: (1) petaloid-shaped hyperautofluorescence in the area of macular edema and (2) hyperautofluorescence coincident with yellow subretinal deposits. (3) Diffuse hyperautofluorescence appeared within the area of serous retinal detachment (SRD) and OCT showed precipitates on the undersurface of the retina in 5/5 of these eyes (100%). (4) The area of vein occlusion showed diffuse hyperautofluorescence after resolution of the retinal bleeding. (5) Hard exudates exhibited hyper- or hypoautofluorescence. OCT indicated that most of the hard exudates with hyperautofluorescence were located on the retinal pigment epithelium. *Conclusions*. Hyperautofluorescence associated with subretinal fluid or hard exudate appeared in the subretinal space. This type of hyperautofluorescence may be attributed to blood cell or macrophages. FAF and OCT are noninvasive modalities that provide additional information regarding macular edema due to BRVO.

## 1. Introduction 

Retinal vein occlusion is a common vascular disease among individuals aged 65 years and older. Risk factors associated with retinal vein occlusion include arterial hypertension, glaucoma, diabetes mellitus, hyperviscosity, and advanced age [1, 2]. Macular edema is a common sequela and is the major cause of visual disturbance associated with branch retinal vein occlusion (BRVO) [3, 4]. Previous studies suggested that serous retinal detachment (SRD) [5–8], hard exudates at the fovea [9, 10], and loss of the photoreceptor layer on optical coherence tomography (OCT) images [11, 12] are related to a poor visual prognosis. These changes damage the neurosensory retina and the retinal pigment epithelium (RPE). OCT allows observation of the morphological changes in the retina caused by BRVO. However, the pathological involvement of macular edema is not fully understood in living human eyes with BRVO.

Fundus autofluorescence (FAF) imaging is a relatively new technology that can be used to characterize eyes with macular disease. FAF is mainly based on lipofuscin in the RPE, which is a residue of phagocytosed photoreceptor outer segments. FAF can be used to detect pathological changes in the RPE [13]. In addition, FAF reflects changes in the amount of macular pigments and photopigments [14, 15] because the results are affected by these pigments. Therefore, FAF can be used to detect RPE abnormalities, macular pigments, photopigments, and macrophages in the subretinal space. The present study evaluated the features of FAF during the course of BRVO and assessed its association with morphological changes of the retina on OCT.

## 2. Patients and Methods

This study followed the tenets of the Declaration of Helsinki. The institutional review board at Fukushima Medical University School of Medicine approved the methods used in this study, including observation using OCT and FAF for eyes with macular and retinal disorders, the retrospective comparative analysis performed in this study, and the intravitreal injections of bevacizumab administered for retinal edema due to ocular disease. Written informed consent was obtained from all patients.

This study was designed as a retrospective observational case series that included 65 eyes from 64 patients (38 women and 26 men) who visited Fukushima Medical University Hospital from August 2008 to November 2010. The average patient age was 67.4 ± 10.1 (mean ± SD) years (range, 40–85 years). The duration of symptoms from the first visit was 3.8 ± 3.5 (mean ± SD) months. Clinical examination for the diagnosis of BRVO included measurement of best-corrected visual acuity (BCVA), refractive error, and intraocular pressure, along with use of slit-lamp biomicroscopy with a contact lens or a noncontact lens, indirect ophthalmoscopy, and digital fluorescein angiography (TRC-50IX/IMAGEnet H1024 system, Topcon, Tokyo, Japan). Fluorescein angiography (FA) was performed at the first visit and in the follow-up period to evaluate retinal circulation when necessary. Eyes were excluded if ocular diseases that caused macular edema, such as diabetic retinopathy, central serous chorioretinopathy, and age-related macular degeneration, were present. OCT scans (Cirrus OCT, Carl Zeiss Meditec, Jena, Germany; 3D-OCT-TM system Topcon, Tokyo, Japan; Spectralis-TM, Heidelberg Engineering, Heidelberg, Germany) were performed with single scans in horizontal and vertical orientations so that the scans regularly passed through the center of the fovea. The regions of interest were also scanned. Central foveal thickness was measured by placing calipers on the screen of the OCT monitor. The status of the inner segment/outer segment junction (IS/OS) was evaluated. The following observations on OCT images were defined as resolution of macular edema; the cystoid change at the fovea disappeared in both the vertical and horizontal slices and the central foveal thickness was less than 250 *μ*m. FAF was measured using a Heidelberg Retina Angiograph 2 (HRA2; Heidelberg Engineering, Heidelberg, Germany). The images were evaluated after processing using an averaging module in the HRA2 software. 

### 2.1. Statistical Analysis

BCVA was measured by the decimal acuity chart and converted to logMAR for statistical analysis. The data obtained were analyzed with frequency and descriptive statistics. 

## 3. Results

Patients were followed for 6–45 months (average, 24.9 months). Superior branch vein occlusion occurred in 50 out of 65 eyes (77%) (Table 1). During the follow-up period, patients were treated with laser photocoagulation (22 eyes; 34%), vitrectomy (20 eyes; 31%), intravitreal bevacizumab injection (8 eyes; 12%), and sub-Tenon triamcinolone injection (4 eyes; 6%). SRDs were observed in 24 of 65 eyes (37%) at the first visit. Cystoid changes on OCT images remained in 34 eyes (51%) at the final visit (Table 2).

### 3.1. Autofluorescence

**Figure 1:**
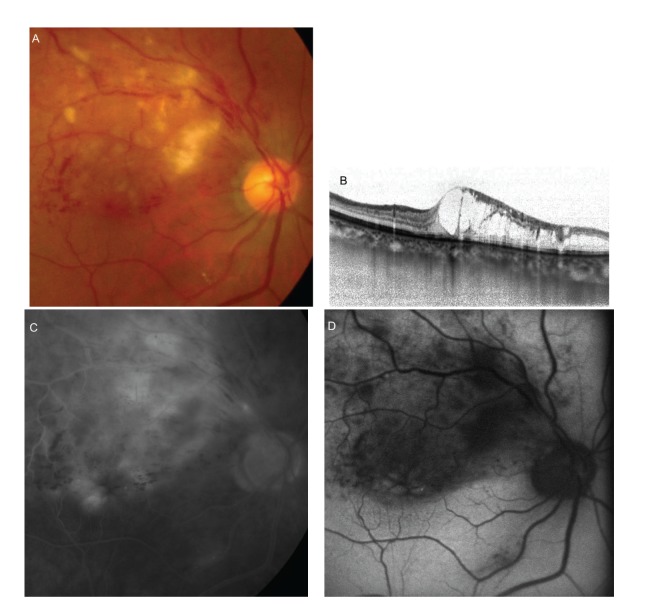
Fundus photograph, OCT, fluorescein angiograph, and autofluorescence images for Patient 1 (a 56-year-old man). A color fundus photograph obtained at the first visit showed an upper temporal vein occlusion in the right eye (A). The best-corrected visual acuity in his right eye was 0.3. A “cotton wool” patch was noticed in the affected area. OCT and fluorescein angiography revealed cystoid macular edema (B). Fluorescein angiography showed a petaloid-shaped hyperfluorescence at the fovea (C). Fundus autofluorescence revealed petaloid autofluorescence corresponding to a cystoid space at the fovea (D).

**Figure 2:**
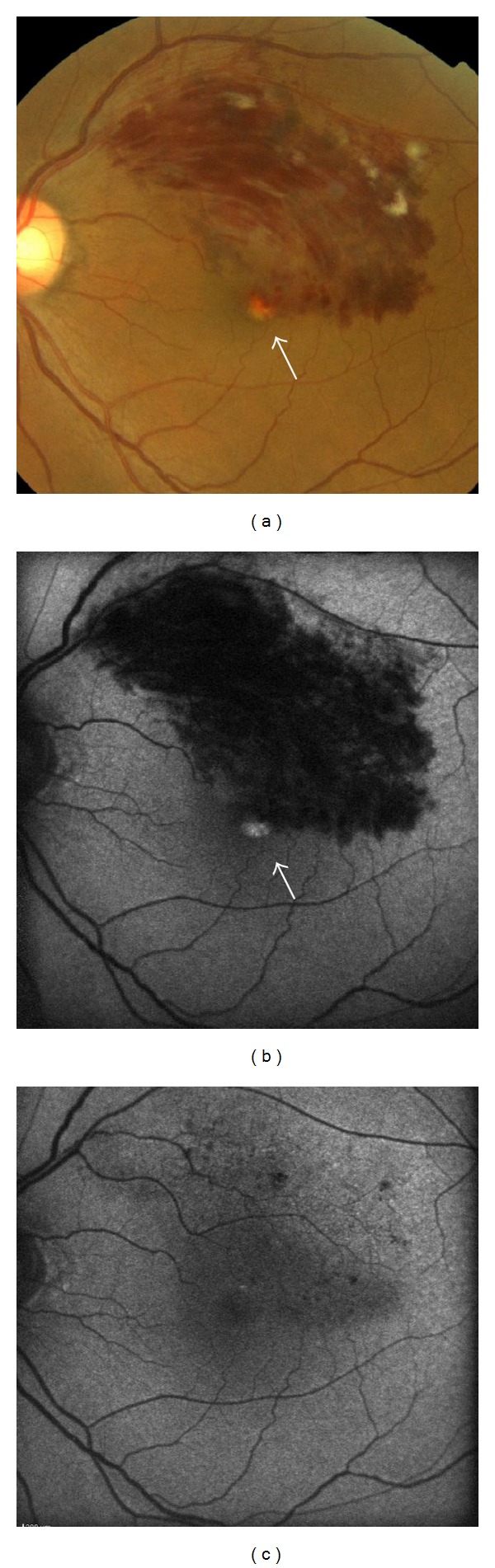
Fundus photograph and fundus autofluorescence images for Patient 5 (a 60-year-old man). Color fundus photograph (a) and fundus autofluorescence (b). Yellowish deposition (white arrow) appeared at the fovea 2 months after the onset of BRVO. Hyperautofluorescence disappeared after resolution of the deposits (c).

**Figure 3:**
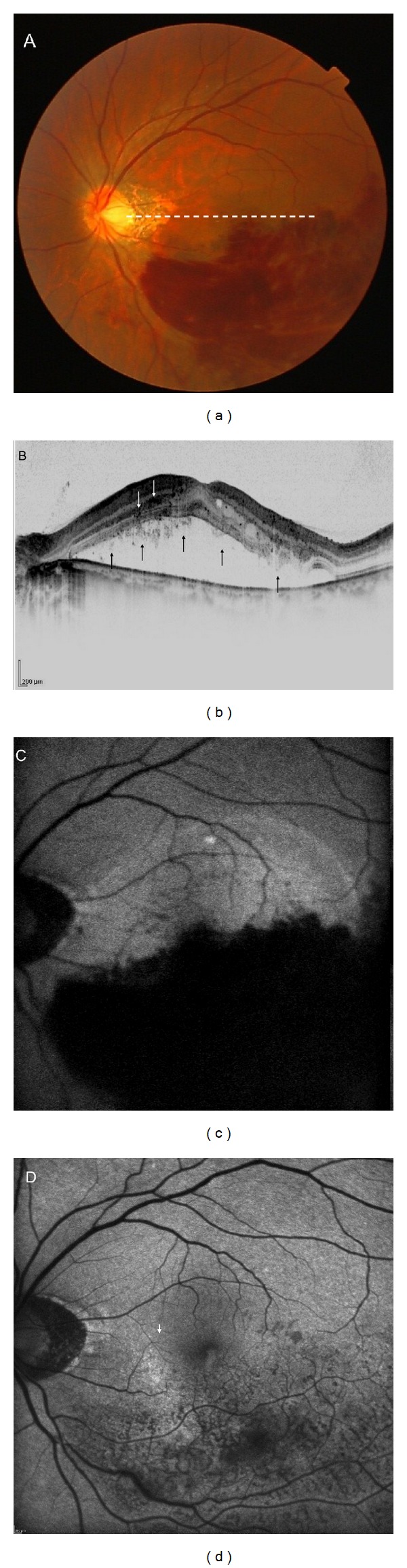
Fundus photograph, OCT, and autofluorescence images for Patient 3 (a 56-year-old man). Color fundus photography at the first visit showed a lower temporal vein occlusion in the left eye (A). The dashed line indicates the OCT scanning line. The OCT image (B) showed widespread deposits on the outer surface of the retina (black arrows). An intraretinal deposit was observed in the outer plexiform layer of the retina (white arrow). Fundus autofluorescence (C) at the first visit showed diffuse hyperautofluorescence in the area of serous retinal detachment. The eye was treated with an intravitreal bevacizumab injection. After the serous retinal detachment was resolved, fundus autofluorescence (D) showed hyperautofluorescence in the nasal area of the fovea (arrowhead). The best-corrected visual acuity in the patient's left eye was 0.7 at the final visit.

**Figure 4:**
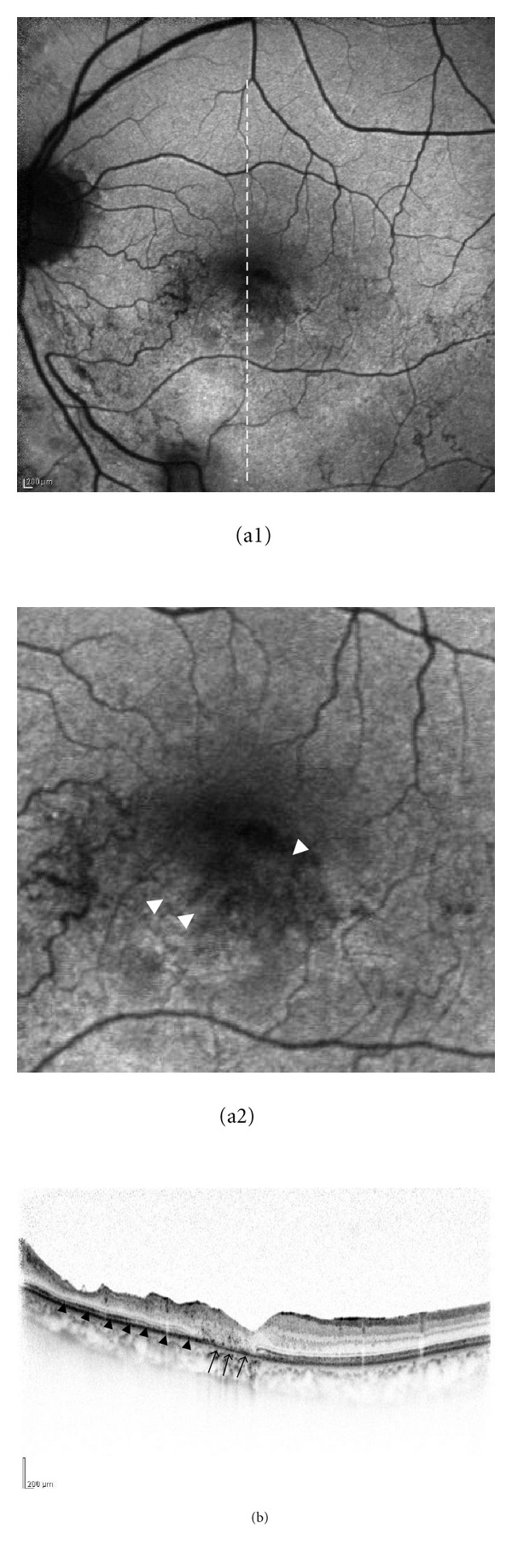
Fundus autofluorescence and OCT images for Patient 4 (a 67-year-old man). Retinal bleeding in the inferotemporal area was resolved 19 weeks after intravitreal bevacizumab injection. (a1) Fundus autofluorescence showed hyperautofluorescence in the inferotemporal area. (a2) High magnification image of the fovea. Hyperautofluorescence appeared in the corresponding area of cystoid macular edema (white arrowheads). The OCT image (b) showed a defect of the inner/outer segments at the fovea (arrows) and thinning of the retina (black arrowheads) corresponding to the area that showed diffuse hyperautofluorescence after resolution of edema (right). The dashed line indicates the OCT scanning line.

**Figure 5:**
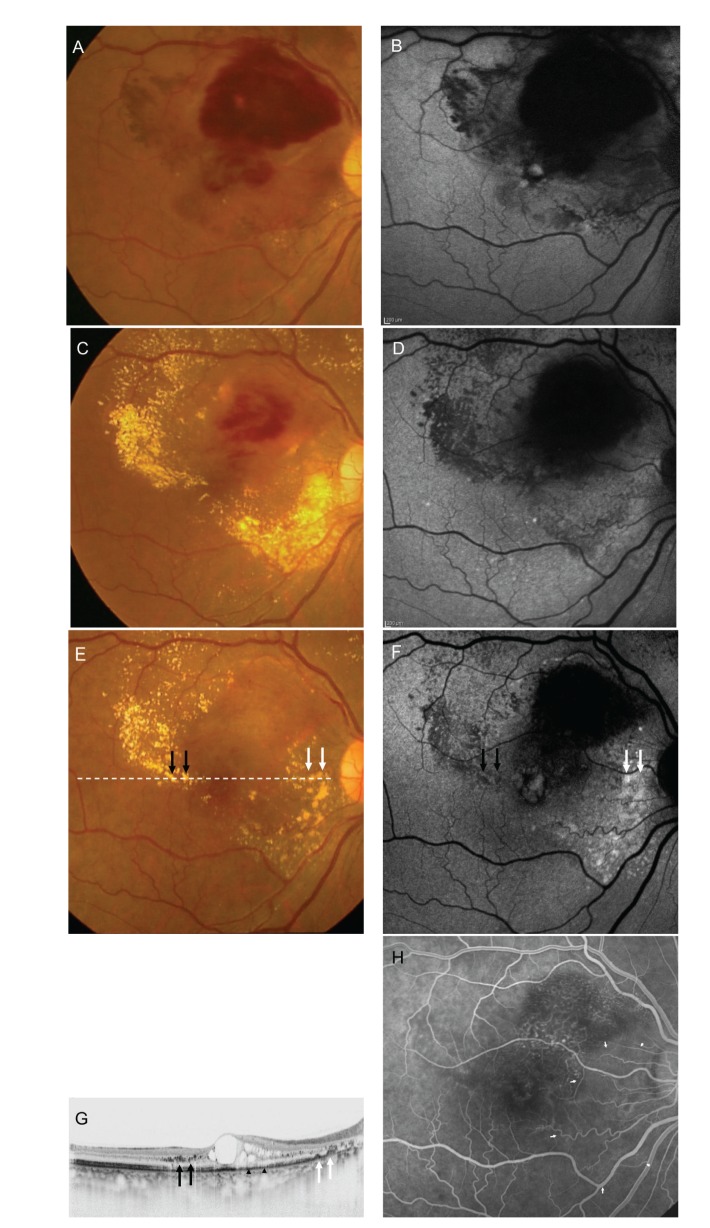
Fundus photograph, fundus autofluorescence, and OCT images for Patient 6 (a 52-year-old woman). The fundus photograph (A) showed a dense retinal hemorrhage and a subretinal hemorrhage. Fundus autofluorescence (B) showed petaloid hyperautofluorescence at the fovea. Extensive exudates were noted 4 months after the first visit (C). Hyperautofluorescence corresponding to the exudates was not observed (D). The color of the exudates in the foveo-papillary area turned yellowish 7 months after the first visit (E). The dashed line indicates the OCT scanning line. Fundus autofluorescence (F) showed hyperautofluorescence in the area corresponding to the yellowish exudates in the foveo-papillary area (white arrows). The exudates in the temporal area (black arrows) showed hypoautofluorescence. OCT (G) at the same visit showed deposits on the retinal pigment epithelium (white arrows) in the foveo-papillary area and intraretinal deposits (white arrows) in the temporal area. The IS/OS disappeared (arrowheads). Fluorescein angiography (H) did not reveal abnormalities in the area corresponding to the area of hyperautofluorescence (white arrowheads).

During the BRVO follow-up period, five types of autofluorescence patterns appeared. The first 2 were at the fovea: (1) petaloid-shaped hyperautofluorescence in the area of macular edema (Figure 1), and (2) hyperautofluorescence coincident with yellow subretinal deposition (Figure 2). In addition, there were cases with diffuse hyperautofluorescence within the area of SRD, in the area of vein occlusion after the resolution of retinal bleeding (Figures 3 and 4), and in association with hard exudates, which showed hyper- or hypoautofluorescence (Figure 5).

### 3.2. Changes in Autofluorescence

At the first visit, OCT showed cystoid change at the fovea in all cases. Petaloid hyperautofluorescence corresponding to the cystoid space at the fovea appeared in 55 of 65 eyes (85%) (Table 3). Cystoid change disappeared in 49 of 65 eyes (49%) by the final visit. Twenty-two of 34 eyes (45%) showed hyperautofluorescence at the fovea after the resolution of macular edema on OCT images (Figure 4). Occasionally hyperautofluorescence aligned radially around the center of the fovea. Some eyes showed hyperautofluorescence only at the center of the fovea. Hyperautofluorescence corresponded to the area that showed IS/OS defect or defect of the outer nuclear layer (Figure 3). Intense hyperautofluorescence coincident with subretinal deposits was observed in 6 of 65 eyes (9%). These eyes frequently showed dense retinal bleeding and SRD. Hyperautofluorescent deposition disappeared during the follow-up period, and no visible changes remained after the resolution of macular edema (Figure 2). Diffuse hyperautofluorescence within the area of the SRD appeared in 5 of 24 eyes with SRD (6%) at the first visit, and OCT revealed precipitates on the undersurface of the retina in all of these eyes (5/5, 100%). Diffuse hyperautofluorescence was clearly observed when the SRD extended beyond the BRVO-affected area. This type of autofluorescence persisted within the area of the SRD for several weeks after resolution of the SRD (Figure 3). After the resolution of retinal hemorrhage, diffuse hyperautofluorescence appeared in the area of the vein occlusion, although the eyes did not show SRDs during the follow-up period. This type of hyperautofluorescence was observed in 19 eyes (20%), and OCT revealed a thin outer retina in the area of diffuse hyperautofluorescence (Figure 4). Hard exudates showed hypo- or hyperautofluorescence (Figure 5). Of 19 eyes with hard exudates at the first visit, 11 eyes (58%) showed hypoautofluorescence, 1 eye (5%) showed hyperautofluorescence, and 7 eyes (33%) showed mixed hyper- and hypoautofluorescence at the first visit. Of 18 eyes with hard exudates at the final visit, 7 eyes (39%) showed hypoautofluorescence, 3 eyes (17%) showed hyperautofluorescence, and 8 eyes (44%) showed mixed autofluorescence. The incidence of hard exudates with hyperautofluorescence increased during the follow-up period (Table 3). OCT revealed that most of the hard exudates with hyperautofluorescence were located on the RPE (Figure 5).

## 4. Discussion

In the current study, autofluorescence appearing in eyes with BRVO and macular edema was uniformly observed in association with OCT findings. Eyes with BRVO showed petaloid autofluorescence, diffuse autofluorescence, autofluorescence associated with SRD, diffuse hyperautofluorescence after resolution of retinal bleeding, and autofluorescence coincident with hard exudate. 

Previous reports have described autofluorescence related to macular edema in retinal vascular disease including BRVO [14, 16–18]. In general, eyes with macular edema showed petaloid autofluorescence that resulted from a reduction of blockage by macular pigments in the area of the cystoid space [14]. Hyperautofluorescence appeared after resolution of the macular edema. Because the IS/OS at the fovea disappeared in these eyes, hyperautofluorescence was attributed to defects of photoreceptor cells at the area of the cystoid space. Hyperautofluorescence at the fovea, which is associated with a decrease in the photoreceptor outer segments, was reported in eyes after macular hole surgery [19]. Intense hyperautofluorescence with subretinal deposits appeared in eyes with dense retinal hemorrhage. Since blood cells show hyperautofluorescence in the subretinal space [20], this type of autofluorescence may have originated from subretinal blood cells. 

So-called hard exudates are often seen in eyes with retinal vascular diseases such as diabetic retinopathy, hypertensive retinopathy, and retinal vein occlusion. These exudates are composed of lipid and proteinaceous material such as fibrinogen and albumin [21, 22]. Hard exudates and lipid deposits show hypoautofluorescence in general [23]. In the current study, hard exudates showed hypo- or hyperautofluorescence in eyes with BRVO, and most of the hard exudates in the subretinal space showed hyperautofluorescence. Autofluorescence may be generated by an interaction between the constituents of exudates, the outer segment of the photoreceptors, and the cells in the subretinal space. We speculated 2 mechanisms to explain autofluorescence of hard exudates in the subretinal space. The first mechanism is the chemical reaction of iron in the subretinal space. The iron in subretinal clots presumably produces autofluorescent compounds that mediate iron-catalyzed free-radical attacks on the lipids of the photoreceptor outer segments through the Fenton reaction [20, 24]. This reaction, which may induce damage to the neurosensory retina and the RPE, may occur in the subretinal space if hard exudates contain iron. The second mechanism is the accumulation of macrophages in the RPE. Hard exudates contain macrophages [21], and macrophages migrate into the subretinal fluid and generate autofluorescence in exudative retinal detachments, such as with Coats' disease [25]. Therefore, the accumulation of macrophages around exudates can cause autofluorescence. The above 2 mechanisms can be involved in the intense autofluorescence coincident with deposits at the fovea. 

The areas with SRDs showed diffuse hyperautofluorescence, which was previously reported in eyes with central serous chorioretinopathy [25–28]. These reports suggested that fragments of the photoreceptor outer segment or macrophages could also cause diffuse autofluorescence. Our study showed that the evidence of deposits on OCT supports the infiltration of cells on the outer retinal surface. Macrophages may be attributed to diffuse hyperautofluorescence within the SRD in a way analogous to central serous chorioretinopathy. This type of autofluorescence can be involved in blood cells for the same reasons as hyperautofluorescence with a hard exudate. 

A decrease in photoreceptor layer thickness was reported to increase FAF [29, 30]. OCT revealed a decrease in retinal thickness in the areas showing diffuse hyperautofluorescence after the resolution of retinal bleeding associated with BRVO. Therefore, this pattern of hyperautofluorescence suggests severe damage to the outer retina. 

This study had several limitations with respect to clinical observations. The relationship between the origin of autofluorescence and the histopathological findings was not studied. Furthermore, the cells and materials that generated the autofluorescence were not identified conclusively. Further investigation of FAF should address these limitations. 

## 5. Conclusion

Eyes with BRVO showed petaloid autofluorescence, diffuse autofluorescence, and autofluorescence coincident with deposits and exudate. Decreases in macular pigments and photoreceptor outer segments cause hyperautofluorescence of the fovea. Hyperautofluorescence associated with subretinal fluid or hard exudate appeared in the subretinal space. This type of hyperautofluorescence may be attributed to blood cell components or macrophages. The combination of FAF and OCT can noninvasively provide additional information about macular edema due to BRVO.

## Figures and Tables

**Table 1:** Patient characteristics.

	*N*	
Sex		
Female	39	60%
Male	26	40%
Laterality		
Left	25	38%
Right	40	62%
Location of BRVO		
Inferior	15	23%
Superior	50	77%

Duration (mean ± SD)	3.8 ± 3.5 months	

Duration: duration of symptoms from the first visit.

**Table 2:** OCT findings.

	At the first visit	At the final visit
Serous retinal detachment	24/65 eyes (37%)	1/65 eyes (2%)
Defect of the IS/OS line	*	32/65 eyes (52%)
Cystoid changes	65/65 eyes (100%)	34/65 eyes (51%)

^∗^Not evaluated.

**Table 3:** Petaloid autofluorescence.

Cystoid change (OCT)		Petaloid hyperautofluorescence				Total
		Yes		No	
At the first visit	Yes	55	(85%)	10	(15%)	65
	No	0	(0%)	0	(0%)	0

At the final visit	Yes	12	(75%)	4	(25%)	16
	No	22	(45%)	27	(65%)	49
